# Development and validation of a prediction model for postoperative urinary retention after prolapse surgery: A retrospective cohort study

**DOI:** 10.1186/s12905-024-03171-3

**Published:** 2024-06-07

**Authors:** Min Ju Kim, Sungyoung Lee, So Yeon Lee, Sumin Oh, Myung Jae Jeon

**Affiliations:** 1 Department of Obstetrics and Gynecology, Kyungpook National University Chilgok Hospital, Daegu, Korea; 2 Department of Genomic Medicine, Seoul National University Hospital, Seoul, Korea; 3 Department of Obstetrics and Gynecology, Gangseo MizMidi Hospital, Seoul, Korea; 4 Department of Obstetrics and Gynecology, Korea University Guro Hospital, Seoul, Korea; 5 Department of Obstetrics and Gynecology, Seoul National University College of Medicine, 103 Daehak-ro, Jongno-gu, Seoul, 03080 Korea; 6 Department of Obstetrics and Gynecology, Seoul National University Hospital, Seoul, Korea

**Keywords:** Clinical decision-making, Pelvic organ prolapse, Urinary retention

## Abstract

**Background:**

Postoperative urinary retention (POUR), a common condition after prolapse surgery with potential serious sequelae if left untreated, lacks a clearly established optimal timing for catheter removal. This study aimed to develop and validate a predictive model for postoperative urinary retention lasting > 2 and > 4 days after prolapse surgery.

**Methods:**

We conducted a retrospective review of 1,122 patients undergoing prolapse surgery. The dataset was divided into training and testing cohorts. POUR was defined as the need for continuous intermittent catheterization resulting from a failed spontaneous voiding trial, with passing defined as two consecutive voids ≥ 150 mL and a postvoid residual urine volume ≤ 150 mL. We performed logistic regression and the predicted model was validated using both training and testing cohorts.

**Results:**

Among patients, 31% and 12% experienced POUR lasting > 2 and > 4 days, respectively. Multivariable logistic model identified 6 predictors. For predicting POUR, internal validation using cross-validation approach showed good performance, with accuracy lasting > 2 (area under the curve [AUC] 0.73) and > 4 days (AUC 0.75). Split validation using pre-separated dataset also showed good performance, with accuracy lasting > 2 (AUC 0.73) and > 4 days (AUC 0.74). Calibration curves demonstrated that the model accurately predicted POUR lasting > 2 and > 4 days (from 0 to 80%).

**Conclusions:**

The proposed prediction model can assist clinicians in personalizing postoperative bladder care for patients undergoing prolapse surgery by providing accurate individual risk estimates.

## Background

Postoperative urinary retention (POUR) is a common complication in women undergoing prolapse surgery, with an incidence of 26-86% [1–5]. Although it is usually temporary, POUR may cause a delayed recovery with prolonged hospital stay and significant anxiety and distress to patients [2, 5, 6]. In addition, unrecognized POUR can lead to serious sequelae including urinary tract infection (UTI), detrusor dysfunction, and even damage to surgical repair [7]. Therefore, all women undergoing prolapse surgery need bladder drainage in the perioperative period, usually with the use of indwelling catheters [7]. However, the optimal timing of catheter removal has not been clearly established.

Currently, the majority of urogynecologists remove the indwelling catheter within 2 days postoperatively [8]. However, a systematic review found that early catheter removal (≤ 2 days) was associated with a reduced incidence of UTI and length of hospital stay but an increased risk of recatheterization compared with later catheter removal (> 2 days) after prolapse surgery [9]. As recatheterization is often considered the worst part of the surgical experience and even a surgical complication for patients [10–12], the preferable timing of catheter removal needs to be viewed from the patient’s perspective. One recent study showed that the mean time to return of bladder function after native tissue vaginal reconstruction was 4.1 days, with one-third of patients experiencing POUR beyond 4 days [13]. Given that postoperative bladder function may be influenced by various clinical and surgical factors [7], providing an individual risk estimate through a prediction model integrating these factors might be useful to guide the optimal timing of catheter removal.

The aim of this study was to develop and validate a prediction model for urinary retention lasting > 2 and > 4 days after prolapse surgery.

## Methods

We reviewed the medical records of 1,122 Korean women who underwent prolapse surgery in a tertiary hospital in South Korea between October 2008 and February 2022. Among them, 82 patients who underwent intraoperative bladder injury repair (*n* = 21), could not undergo a spontaneous voiding trial (e.g., oliguria from end-stage renal disease, history of urinary diversion surgery, *n* = 3), had incomplete data regarding voided volume and postvoid residual (PVR, *n* = 32), or received reinsertion of an indwelling transurethral catheter instead of intermittent catheterization after an unsuccessful initial voiding trial (*n* = 26) were excluded from the analyses. The study was approved by the institutional review board (Seoul National University College of Medicine/Seoul National University Hospital 2207-078-1339).

All patients underwent a spontaneous voiding trial on postoperative day (POD) 1 or 2. An indwelling transurethral catheter was removed, and the bladder was allowed to fill spontaneously over no more than 4 h. Patients were then instructed to void as needed into a measuring container, after which straight catheterization was performed to assess PVR. Patients who had two consecutive voids ≥ 150 mL with a PVR ≤ 150 mL were considered to have passed the voiding trial [14]. Patients who failed the voiding trial were offered intermittent catheterization until they had two consecutive PVRs of ≤ 150 mL. POUR was defined as the need for continuous intermittent catheterization resulting from a failed voiding trial.

Based on a review of the literature [7], the following baseline demographic and clinical characteristics were selected as candidate predictors for POUR: age (years), body mass index (kg/m^2^), diabetes mellitus, pelvic organ prolapse quantification stage, preoperative PVR (mL), type of surgery for apical prolapse, concomitant hysterectomy, anterior repair (AR), posterior repair (PR) and midurethral sling (MUS), intraoperative estimated blood loss (mL) and operation time (min). Type of anesthesia, volume of intraoperative fluid administration and postoperative UTI were not included because all patients underwent surgery under general anesthesia and had been catheterized during the operation to avoid bladder overdistension, and postoperative UTI is usually perceived as a consequence rather than a cause of POUR. The pelvic organ prolapse quantification examination was performed in a 45° upright sitting position with an empty bladder and preoperative PVR was measured by catheterization. The type of surgery for apical prolapse was classified as intraperitoneal native tissue apical suspension (high uterosacral ligament suspension, vaginal or laparoscopic), extraperitoneal native tissue apical suspension (sacrospinous ligament fixation or iliococcygeus suspension), sacrocolpopexy with mesh and colpocleisis. MUS included both retropubic and transobturator midurethral slings, with the latter being mostly used.

In statistical analyses, we utilized the R programming language and its packages. Ahead of the main analysis, the dataset was split into two parts (training and testing cohorts) with a 2:1 ratio, ensuring the balance of POUR status was maintained. Using the training cohort, we constructed the prediction model and conducted internal validation. A multivariable logistic regression using both exhaustive and stepwise variable selection was performed to construct a prediction model, as described in the previous study [15]. Missing risk factor values were assessed for missing at random, and multiple imputation by chained equations algorithm was applied to estimate missing values. Internal validation was performed using five- and ten-fold cross-validation and their performance was compared to check overfitting. For split validation, the fitted model from the training cohort was applied to the testing cohort. Model calibration was visually performed using the calibration plot. Finally, the performance of model was measured using the area under the receiver operating characteristic curve (AUC).

## Results

The proportion of patients who experienced POUR > 2 days and > 4 days were 31% and 12%, respectively. The baseline characteristics of the training (*n* = 695) cohort and testing (*n* = 345) cohort are summarized in Table 1. There were no significant differences between the two cohorts. The preoperative PVR results were missing in 262 (25%) patients because they did not undergo the test. There were no missing data on other variables.

**Table 1:** Baseline characteristics of the training and testing cohorts

Characteristics	Total (*n* = 1040)	Training (*n* = 695)	Testing (*n* = 345)	*P*-value
Age, years	67.0 (61.0–73.0)	67.0 (61.0–73.0)	67.0 (61.0–72.0)	0.633
Body mass index, kg/m^2^	24.8 (22.9–26.8)	24.7 (22.9–26.8)	24.9 (22.9–26.8)	0.357
Diabetes	174 (16.7)	115 (16.5)	59 (17.1)	0.821
POPQ stage				0.669
2	201 (19.3)	135 (19.4)	66 (19.1)	
3	676 (65.0)	456 (65.6)	220 (63.8)	
4	163 (15.7)	104 (15.0)	59 (17.1)	
Preoperative PVR > 150 mL^a^	89/778 (11.4)	62/523 (11.9)	27/255 (10.6)	0.602
Surgery for apical prolapse				0.688
NTR (USLS)	482 (46.3)	316 (45.5)	166 (48.1)	
NTR (SSLF, ICG)	113 (10.9)	73 (10.5)	40 (11.6)	
SCP	377 (36.3)	258 (37.1)	119 (34.5)	
Colpocleisis	68 (6.5)	48 (6.9)	20 (5.8)	
Concomitant hysterectomy	748 (71.9)	498 (71.7)	250 (72.5)	0.785
Concomitant anterior repair	321 (30.9)	218 (31.4)	103 (29.9)	0.619
Concomitant posterior repair	607 (58.4)	408 (58.7)	199 (57.7)	0.752
Concomitant MUS	419 (40.3)	282 (40.6)	137 (39.7)	0.789
Estimated blood loss, mL	150 (100–220)	150 (100–210)	150 (100–235)	0.282
Operation time, min	160 (125–205)	160 (130–205)	155 (120–200)	0.378

ICG, iliococcygeus suspension; MUS, midurethral sling; NTR, native tissue repair; POPQ, pelvic organ prolapse quantification; PVR, postvoid residual; SCP, sacrocolpopexy; SSLF, sacrospinous ligament fixation; USLS, uterosacral ligament suspension

Data are presented as median (interquartile range) or number (%)

^a^ Preoperative PVR results were not available for 262 cases, and they were not included as a denominator

**Fig. 1:**
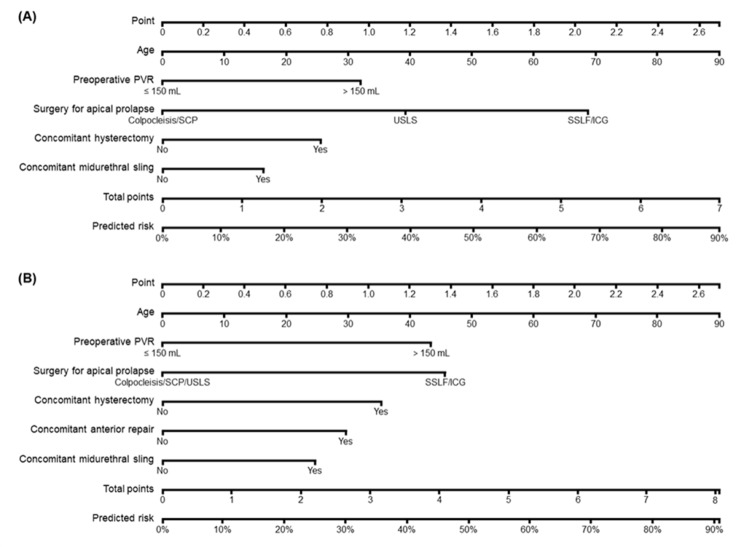
Nomogram for predicting the risk of postoperative urinary retention > 2 days (**A**) and > 4 days (**B**). ICG, iliococcygeus suspension; PVR, postvoid residual; SCP, sacrocolpopexy; SSLF, sacrospinous ligament fixation; USLS, uterosacral ligament suspension

Using the training cohort, our multivariable logistic model with exhaustive variable selection identified six predictors for the model: age, preoperative PVR, type of surgery for apical prolapse, concomitant hysterectomy, AR and MUS. The stepwise selection also selected the same variables with the addition of body mass index (for the model lasting > 2 days) and operation time (for the model lasting > 4 days). The exhaustive model had a slightly higher AUC than the stepwise model and was selected as the final model. Increasing age, elevated preoperative PVR (> 150 mL), native tissue apical suspension, concomitant hysterectomy, and MUS had incremental effects on POUR lasting > 2 days. With the exception of uterosacral ligament suspension, all these variables had incremental effects on POUR lasting > 4 days, and concomitant AR also increased the risk of POUR lasting > 4 days (Table 2). Figure 1 presents the nomogram using the reference model with these predictors.

**Table 2:** Risk factors and their estimated contribution to the logistical model for predicting urinary retention after prolapse surgery

Variables	OR (95% CI)	
	Model for POUR > 2 days	Model for POUR > 4 days
Age (per year)	1.04 (1.02–1.05)	1.03 (1.00-1.05)
Preoperative PVR > 150 mL	2.61 (1.66–4.12)	3.66 (2.13–6.18)
NTR (USLS)	3.24 (2.31–4.60)	NA
NTR (SSLF, ICG)	7.86 (4.88–12.81)	3.93 (2.23–6.91)
Concomitant hysterectomy	2.16 (1.48–3.19)	2.90 (1.66–5.31)
Concomitant anterior repair	NA	2.43 (1.57–3.75)
Concomitant MUS	1.63 (1.22–2.19)	2.09 (1.39–3.16)

CI, confidence interval; ICG, iliococcygeus suspension; OR, odds ratio; MUS, midurethral sling; NA, not available; NTR, native tissue repair; POUR, postoperative urinary retention; PVR, postvoid residual; SSLF, sacrospinous ligament fixation; USLS, uterosacral ligament suspension

Logistic regression equation of model for POUR > 2 days: -4.87 + 0.03 × Age + 0.96 × Preoperative PVR > 150 mL + 1.18 × NTR (USLS) + 2.06 × NTR (SSLG, ICG) + 0.77 × Concomitant hysterectomy + 0.49 × Concomitant midurethral sling

Logistic regression equation of model for POUR > 4 days: -5.76 + 0.03 × Age + 1.30 × Preoperative PVR > 150 mL + 1.37 × NTR (SSLG, ICG) + 1.06 × Concomitant hysterectomy + 0.89 × Concomitant anterior repair + 0.74 × Concomitant midurethral sling

**Fig. 2:**
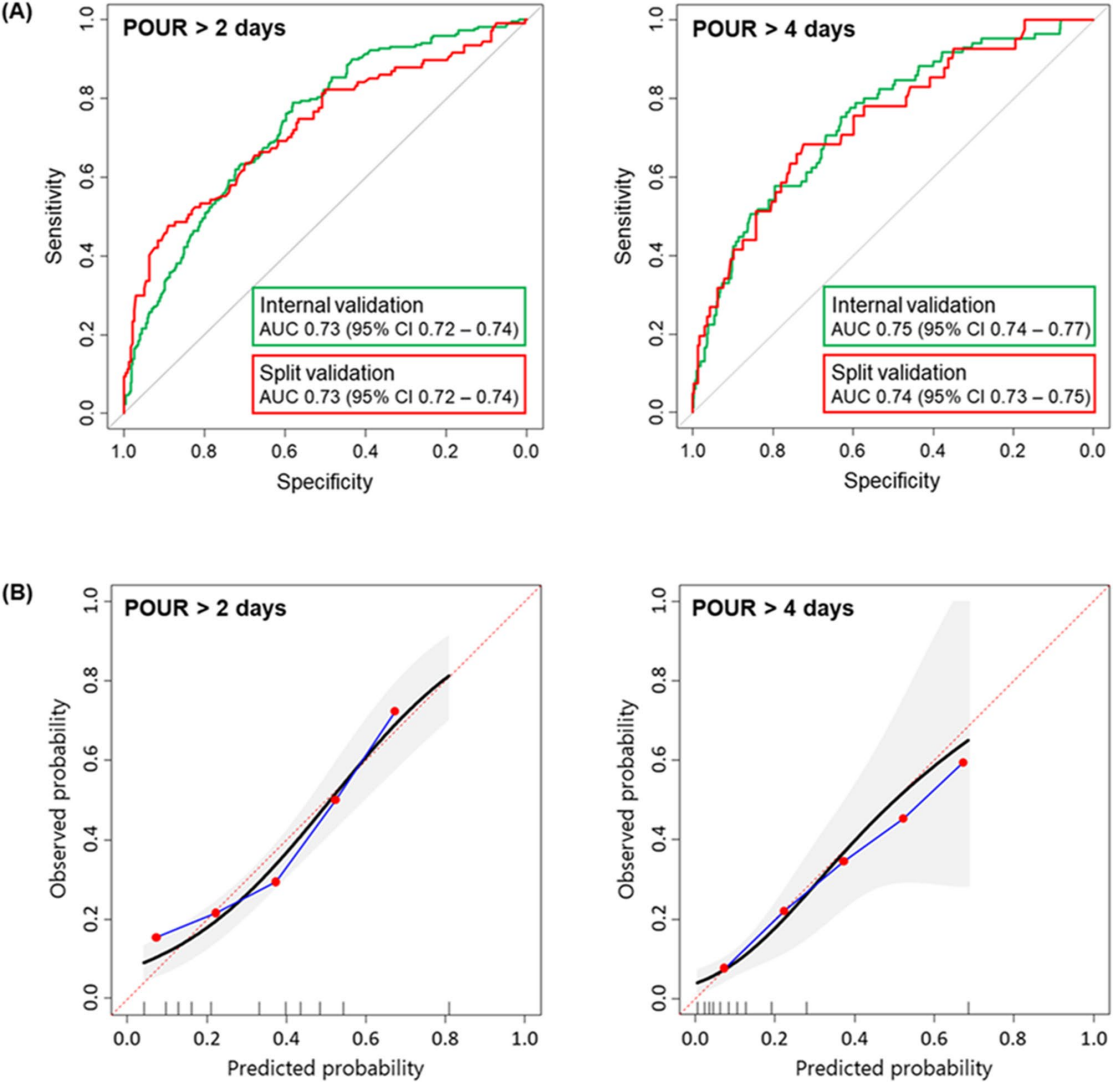
(**A**) Prediction performance of the proposed model. Internal validation using five-fold cross-validation (green) and split validation using the testing cohort (red). AUC, area under the curve; CI, confidence interval; POUR, postoperative urinary retention. (**B**) Calibration curve of the prediction model. Dots indicate observed probabilities of each bin, and the blue line represents the calibration curve. The grey shading indicates 95% confidence intervals. POUR, postoperative urinary retention

We next conducted validation of the model against both training and testing cohorts. On the training cohort, internal validation using five-fold cross-validation showed good performance for predicting POUR lasting > 2 (AUC 0.73, 95% confidence interval [CI] 0.72–0.74) and > 4 days (AUC 0.75, 95% CI 0.74–0.77). Subsequent analysis using ten-fold cross-validation showed that the model manifests stable prediction power (AUC 0.73 for POUR lasting > 2 days and 0.74 for POUR lasting > 4 days). On the testing cohort, split validation also showed good performance for predicting POUR lasting > 2 (AUC 0.73, 95% CI 0.72–0.74) and > 4 days (AUC 0.74, 95% CI 0.73–0.75) (Fig. 2A). Calibration curves demonstrated that the model accurately predicted the observed outcomes of POUR lasting > 2 and > 4 days (from 0 to 80%) (Fig. 2B).

## Discussion

In this study, we identified six predictors (age, preoperative PVR, type of surgery for apical prolapse, concomitant hysterectomy, AR and MUS) and developed a prediction model for POUR by the period after prolapse surgery. This model showed good predictive performance and accurately predicted the observed outcomes. The proposed model is provided as an online risk calculator (http://lsy.io/nomogramPOUR).

There exist three prediction models for POUR following pelvic floor surgery [16–18]. These models provide an individual risk estimate of failure to pass the initial voiding trial on POD 0–2. Although it may be helpful in view of preoperative counseling and managing patient expectations, it cannot guide the optimal timing of catheter removal. Our model provides an individual risk estimate of POUR lasting > 2 and > 4 days, which could be useful in personalizing postoperative bladder care for patients undergoing prolapse surgery.

Consistent with the existing models, several vaginal procedures performed for the correction of pelvic organ prolapse were included in our model as predictors for POUR. This is likely due to pelvic floor tension secondary to pain and neuropathy resulting from the disruption of peripheral pelvic nerve branches involved in bladder sensation and micturition [7]. Our study identified native tissue apical suspension as a risk factor for POUR and is in agreement with recent studies that showed native tissue apical suspension had a three- to fivefold greater risk of acute POUR compared to sacrocolpopexy [19, 20]. Extraperitoneal native tissue apical suspension had a greater and prolonged risk of POUR than intraperitoneal apical suspension. The pathologic mechanism for this difference is not clear but may be related to higher rates of neurologic pain requiring opioid use and concomitant levator ani plication in women receiving extraperitoneal native tissue apical suspension [1, 21]. Unlike AR, PR was not identified as a significant predictor of POUR in our model. PR does not involve manipulation of the bladder or urethra but may impair voiding function by causing pain that prevents relaxation of the pelvic floor muscles, particularly when performed with levator ani plication [1]. We avoided levator ani plication as much as possible except for women receiving extraperitoneal native tissue apical suspension, which may explain why PR was not included as a predictor in the model.

Our study found that concomitant hysterectomy doubles the risk of POUR, which is consistent with recent studies [22–24]. Concomitant MUS was also found to be a risk factor, which was included as a significant predictor variable in one previous model [17] but not in the other two models [16, 18]. Although this discrepancy may be related to variations in sling tensioning, it may also be due to the difference in the study populations used for model development (training cohort). All of the patients in our study population underwent prolapse surgery, whereas many women who had undergone only anti-incontinence surgery were included in other existing models. A recent systematic review also reported that concomitant MUS at the time of prolapse surgery increased the risk of POUR [25].

Apart from surgical procedures, we also identified some clinical and demographic factors that were associated with POUR. Older age had an incremental effect on POUR as reported in many previous studies [18, 19, 26], which may be associated with age-related neuronal degeneration leading to bladder dysfunction [27]. Baseline bladder dysfunction was also identified as a significant risk factor for POUR in our model. Consistently, elevated PVR was included as a risk factor in all existing models except one model that did not include it as a candidate variable [16–18].

With the concept of enhanced recovery after surgery (ERAS) gaining popularity, early catheter removal has become a clinical trend. The American Urogynecologic Society and International Urogynecologic Association Joint clinical consensus statement on ERAS after urogynecologic surgery also recommends that the catheter be removed as soon as feasible once there is no clinical necessity [28]. Several randomized controlled trials and a systematic review of these trials showed that early catheter removal (on POD 1–2) is more advantageous than later removal (on POD 3–5), with a lower incidence of UTI and a shorter hospital stay, although it is associated with an increased risk of recatheterization [9, 29–31]. However, these trials either did not include or had a small number of patients who underwent native tissue apical suspension. Another randomized controlled trial found that women who had an unsuccessful same-day voiding trial after vaginal reconstructive surgery including native tissue apical suspension had a 7-fold higher risk of an unsuccessful repeat voiding trial when the repeat trial was performed within 4 days after surgery than when performed on POD 7. The rates of UTI were also higher in the earlier repeat voiding trial group [32].

Our prediction model provides an individual risk estimate of POUR lasting > 2 and > 4 days. This information may be useful in determining the optimal timing of catheter removal, especially when the patients are unable to learn self-catheterization or prefer to have an indwelling catheter. For example, in patients with > 50% risk of POUR > 2 days, the indwelling catheter removal needs to be delayed. According to the risk of POUR lasting > 4 days, the timing of catheter removal for these patients can be individualized: on POD 4 (if the risk < 50%) and 7 (if the risk > 50%). The risk estimate calculated from our prediction model will also aid in individualizing a repeat voiding trial in women who failed the initial voiding trial and are discharged with an indwelling catheter.

The current study has several strengths. Our model covers most types of prolapse surgery being performed in current practice, and therefore, it can be applied to most women undergoing prolapse surgery. Unlike other existing models, our model provides risk estimates of POUR by different time periods, which can be useful in personalizing postoperative bladder care. The large sample size enabled the split validation using the testing cohort completely separated from the training cohort, and we confirmed the model’s discriminative ability and accuracy. Furthermore, the availability of an online risk calculator makes this model convenient to use. Nonetheless, this study has some limitations. The retrospective study design did not allow complete data collection, and preoperative PVR results were missing in 25% of patients. Instead of excluding eligible patients due to missing data, missing values were imputed using multiple imputation for model construction. In addition, information regarding postoperative opioid use, which may impact voiding function, could not be collected. In our institution, opioids are not routinely used for pain control but are used for breakthrough pain when acetaminophen and non-steroidal anti-inflammatory drugs are not sufficient. Second, all procedures were performed by a single surgeon, and patients only had an MUS if they had stress incontinence, which may limit the generalizability of the results. Lastly, it may be arguable whether the proposed model is applicable to populations with different baseline characteristics from ours. The predictive accuracy of our model needs to be validated further in cohorts with different backgrounds.

## Conclusions

We successfully developed and validated a clinical prediction model to calculate the risk of POUR after prolapse surgery. For patients planning to undergo prolapse surgery, our prediction model might be a useful tool for clinicians to personalize postoperative bladder care. Further external validation will be required to verify this model’s utility in clinical practice with different patient characteristics.

## Data Availability

The datasets used and/or analyzed during the current study are available from the corresponding author on reasonable request.

## Abbreviations

POUR: Postoperative urinary retention
UTI: Urinary tract infection
PVR: Postvoid residual
POD: Postoperative day
AR: Anterior repair
PR: Posterior repair
MUS: Midurethral sling
AUC: Area under the curve
CI: Confidence interval
ERAS: Enhanced recovery after surgery

## References

[1] Hakvoort RA, Dijkgraaf MG, Burger MP, Emanuel MH, Roovers JP (2009). Predicting short-term urinary retention after vaginal prolapse surgery. Neurourol Urodyn.

[2] Carter-Brooks CM, Zyczynski HM, Moalli PA, Brodeur PG, Shepherd JP (2018). Early catheter removal after pelvic floor reconstructive surgery: a randomized trial. Int Urogynecol J.

[3] Eto C, Ford AT, Smith M, Advolodkina P, Northington GM (2019). Retrospective cohort study on the Perioperative Risk factors for transient voiding dysfunction after apical prolapse repair. Female Pelvic Med Reconstr Surg.

[4] Alas A, Martin L, Devakumar H, Frank L, Vaish S, Chandrasekaran N, Davila GW, Hurtado E (2020). Anesthetics’ role in postoperative urinary retention after pelvic organ prolapse surgery with concomitant midurethral slings: a randomized clinical trial. Int Urogynecol J.

[5] Anglim BC, Ramage K, Sandwith E, Brennand EA (2021). Postoperative urinary retention after pelvic organ prolapse surgery: influence of peri-operative factors and trial of void protocol. BMC Womens Health.

[6] Gagnon LH, Tang S, Brennand E (2017). Predictors of length of stay after urogynecological surgery at a tertiary referral center. Int Urogynecol J.

[7] Geller EJ (2014). Prevention and management of postoperative urinary retention after urogynecologic surgery. Int J Womens Health.

[8] Marschalek ML, Umek W, Koelbl H, Veit-Rubin N, Bodner-Adler B, Husslein H (2021). Wide variation in post-void residual management after urogynecologic surgery: a Survey of urogynecologists’ practices. J Clin Med.

[9] Xie N, Hu Z, Ye Z, Xu Q, Chen J, Lin Y (2021). A systematic review comparing early with late removal of indwelling urinary catheters after pelvic organ prolapse surgery. Int Urogynecol J.

[10] Elkadry EA, Kenton KS, FitzGerald MP, Shott S, Brubaker L (2003). Patient-selected goals: a new perspective on surgical outcome. Am J Obstet Gynecol.

[11] Mahajan ST, Elkadry EA, Kenton KS, Shott S, Brubaker L (2006). Patient-centered surgical outcomes: the impact of goal achievement and urge incontinence on patient satisfaction one year after surgery. Am J Obstet Gynecol.

[12] Kenton K, Pham T, Mueller E, Brubaker L (2007). Patient preparedness: an important predictor of surgical outcome. Am J Obstet Gynecol.

[13] Hines KN, McKenzie C, Overholt T, Mirzazadeh M, Matthews CA, Schachar J, Russel G, Lentz S (2021). Predicting the return of bladder function following vaginal native tissue repair using data from a suprapubic catheter regimen. Neurourol Urodyn.

[14] Koh N, Kim MJ, Lee SY, Oh SM, Jeon MJ (2023). The diagnotistic accuracy of a retrograde voiding trial for restoration of spontaneous voiding function after prolapse and urinary incontinence surgery. J Minim Invasive Gynecol.

[15] Oh S, Lee S, Hwang WY, Suh DH, Jeon MJ (2022). Development and validation of a prediction model for bothersome stress urinary incontinence after prolapse surgery: a retrospective cohort study. BJOG.

[16] Li ALK, Zajichek A, Kattan MW, Ji XK, Lo KA, Lee PE (2020). Nomogram to predict risk of postoperative urinary Retention in Women undergoing pelvic reconstructive surgery. J Obstet Gynaecol Can.

[17] Zhang BY, Wong JMH, Lee NAK, Geoffrion T (2021). Risk factors for urinary retention after urogynecologic surgery: a retrospective cohort study and prediction model. Neurourol Urodyn.

[18] Anglim BC, Tomlinson G, Paquette J, McDermott CD (2022). A risk calculator for postoperative urinary retention (POUR) following vaginal pelvic floor surgery: multivariable prediction modelling. BJOG.

[19] Yune JJ, Cheng JW, Wagner H, Kim J, Hardesty JS, Siddighi S (2018). Postoperative urinary retention after pelvic organ prolapse repair: vaginal versus robotic transabdominal approach. Neurourol Urodyn.

[20] El Haraki AS, Burns J, Crafton CL, Parker-Autry C, Matthews CA (2022). Voiding function after sacrocolpopexy versus native tissue transvaginal repair for apical pelvic organ prolapse in an ERAS era: a retrospective cohort study. Int Urogynecol J.

[21] Barber MD, Brubaker L, Burgio KL, Richter HE, Nygaard I, Weidner AC, Menefee SA, Lukacz ES, Norton P, Schaffer J (2014). Comparison of 2 transvaginal surgical approaches and perioperative behavioral therapy for apical vaginal prolapse: the OPTIMAL randomized trial. JAMA.

[22] Behbehani S, Delara R, Yi J, Kunze K, Suarez-Salvador E, Wasson M (2020). Predictors of postoperative urinary Retention in Outpatient minimally invasive hysterectomy. J Minim Invasive Gynecol.

[23] Misal M, Behbehani S, Yang J, Wasson MN (2020). Is hysterectomy a risk factor for urinary Retention? A Retrospective Matched Case Control Study. J Minim Invasive Gynecol.

[24] Bekos C, Morgenbesser R, Kölbl H, Husslein H, Umek W, Bodner K, Bodner-Adler B (2020). Uterus Preservation in Case of Vaginal Prolapse surgery acts as a Protector against postoperative urinary Retention. J Clin Med.

[25] van der Ploeg JM, van der Steen A, Oude Rengerink K, van der Vaart CH, Roovers JP (2014). Prolapse surgery with or without stress incontinence surgery for pelvic organ prolapse: a systematic review and meta-analysis of randomised trials. BJOG.

[26] Chong C, Kim HS, Suh DH, Jee BC (2016). Risk factors for urinary retention after vaginal hysterectomy for pelvic organ prolapse. Obstet Gynecol Sci.

[27] Baldini G, Bagry H, Aprikian A, Carli F (2009). Postoperative urinary retention: anesthetic and perioperative considerations. Anesthesiology.

[28] Latthe P, Panza J, Marquini GV, Jankowski CJ, Heisler C, Achtari C, Reagan K, Hickman LC, Haddad J (2022). AUGS-IUGA Joint Clinical Consensus Statement on enhanced recovery after urogynecologic surgery: developed by the Joint Writing Group of the International Urogynecological Association and the American Urogynecologic Society. Individual writing group members are noted in the Acknowledgements section. Urogynecol (Hagerstown).

[29] Hakvoort RA, Elberink R, Vollebregt A, Ploeg T, Emanuel MH (2004). How long should urinary bladder catheterisation be continued after vaginal prolapse surgery? A randomised controlled trial comparing short term versus long term catheterisation after vaginal prolapse surgery. BJOG.

[30] Kamilya G, Seal SL, Mukherji J, Bhattacharyya SK, Hazra A (2010). A randomized controlled trial comparing short versus long-term catheterization after uncomplicated vaginal prolapse surgery. J Obstet Gynaecol Res.

[31] Weemhoff M, Wassen MM, Korsten L, Serroyen J, Kampschöer PH, Roumen FJ (2011). Postoperative catheterization after anterior colporrhaphy: 2 versus 5 days. A multicentre randomized controlled trial. Int Urogynecol J.

[32] Schachar JS, Ossin D, Plair AR, Hurtado EA, Parker-Autry C, Badlani G, Davila GW, Matthews CA (2020). Optimal timing of a second postoperative voiding trial in women with incomplete bladder emptying after vaginal reconstructive surgery: a randomized trial. Am J Obstet Gynecol.

